# Probable Lewy Body Dementia with a Predilection for Auditory Hallucinations

**DOI:** 10.7759/cureus.2602

**Published:** 2018-05-10

**Authors:** Hussam Hindi, James L Lawrence

**Affiliations:** 1 West Virginia School of Osteopathic Medicine, Frederick Memorial Hospital, Lewisburg, USA

**Keywords:** lewy, body, dementia, auditory, hallucinations, antipsychotics

## Abstract

The patient was a 60-year-old male who initially presented to the emergency room with extreme agitation aggravated by internal stimuli as well as visual hallucinations, paranoia, and grandiose delusions. He was diagnosed with nonspecific schizophrenia and treated with risperidone, trazodone, and lithium. Approximately 16 months later, he was readmitted to the hospital trying to enter a stranger’s car that he thought was his. He was confused and was unable to give an accurate history. His psychomotor retardation and confusion were thought to be due to risperidone. When his dose was decreased, he displayed involuntary movements of the mouth and extremities, restlessness, and a patting of his head. The risperidone was switched to zyprexa in an effort to decrease extrapyramidal symptoms. About two months later, he was found unresponsive and catatonic in his car. During his stay, the patient had a fluctuation in the latency of his responses on a daily basis. He would be aware of where he was but was unable to explain why or for how long. He had a difficult time remembering names and was still exhibiting abnormal involuntary movements around the mouth and extremities. The patient’s course of initial predominantly auditory and visual hallucinations that progressed to extrapyramidal symptoms and fluctuating cognition one year later may suggest Lewy body dementia.

## Introduction

Lewy body dementia is a relatively new pathology that was only fully recognized as a diagnosis approximately 25 years ago as a result of improved neuropathological staining that allowed the identification of Lewy bodies on brain autopsy tissue [1]. It is a common form of dementia in the elderly population that is often mistaken for Alzheimer’s dementia or schizophrenia due to the unique and progressive presentation of the disease. Lewy body dementia often presents with visual hallucinations followed by extrapyramidal symptoms and fluctuating levels of cognition. Here, we explore a case of the progressive development of clinical symptoms consistent with the diagnosis of probable Lewy body dementia with a predilection for auditory hallucinations.

## Case presentation

The patient is a 60-year-old male who initially presented to the emergency room in 2016 with a long history of perceptual disturbances, mood symptoms, and religious preoccupation. He was brought to the emergency department due to extreme agitation aggravated by internal stimuli of women professing their love to him. The voices were so overwhelming that, at times, he was unable to sleep. He also presented with visual hallucinations. He was very talkative and grandiose. He described that he will build a church for the homeless and put them to work. His speech was circumstantial and poorly goal-directed. His mini mental exam was a 30/30. His temperature was 97.7°F; pulse was 86/min; respiratory rate was 18/min; blood pressure was 100/55 mm Hg. His initial labs showed a white blood cell (WBC) count of 5.92 THOU/uL; hemoglobin of 15.4 g/dL; hematocrit of 43.6%; platelet count of 203 THOU/uL; sodium of 137 mmol/L; potassium of 4.1 mmol/L; creatinine of 1.0 mg/dL; glucose of 96 mg/dL; calcium of 9.7 mg/dL; aspartate aminotransferase (AST) of 12 U/L; alanine aminotransferase (ALT) of 11 U/L; thyroid-stimulating hormone (TSH) of 1.55 uIU/mL. The urine toxicology screen was negative and rapid plasma reagin (RPR) was nonreactive. The physical exam was unremarkable besides the mood, hallucination, and paranoia symptoms. He was believed to have unspecified schizophrenia, bipolar disorder with psychotic features, or schizotypal personality disorder. He was started on risperidone 0.5 mg BID and was increased to 2 mg BID. Trazodone was initiated for sleep and lithium 300 mg BID for mood stabilization was added throughout the course. His condition improved and he was discharged.

Approximately 16 months later, he was brought in by emergency petition because he was trying to enter a stranger’s car. He mistook the stranger’s car for his own. Upon admission, the patient was unable to provide an accurate history due to confusion and memory impairment. He reported no new visual or auditory hallucinations after being discharged in 2016. He also responded to most questions with “I don't remember.” Vitals at the time were temperature: 97.6 °F; pulse: 96/min; respiratory rate: 18/min; and blood pressure: 97/62 mm Hg. Initial laboratory tests showed WBC count: 6.7 THOU/uL; hemoglobin: 14.3 g/dL; hematocrit: 40.7%; platelet count: 215 THOU/uL; sodium: 128 mmol/L; potassium: 2.8 mmol/L and 4.2 mmol/L when repeated; creatinine: 0.7 mg/dL; glucose: 95 mg/dL; calcium: 8.7 mg/dL; AST: 12 U/L; ALT: 10 U/L; TSH: 1.9 uIU/mL; urine toxicology screen: normal; and RPR: negative. He presented with reduced psychomotor activity and organized thinking as opposed to last years' visit. His prior discharge medication was started and 1 mg of Lorazepam PRN was added for anxiety. During this hospital stay, confusion and psychomotor retardation were noted. In response, his risperidone dose was decreased with no change in psychotic symptoms; however, he began developing extrapyramidal side effects. He displayed involuntary movements of the mouth, flailing movements, restlessness, and a patting of his head. Risperidone was replaced by zyprexa due to the decreased likelihood of extrapyramidal symptoms. After observation, the patient was discharged from the hospital.

**Figure 1:**
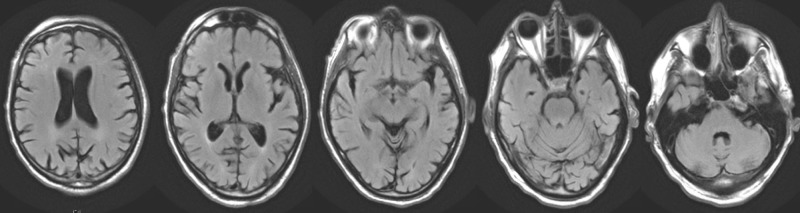
T2 Flair Propeller T2 flair propeller magnetic resonance imaging (MRI) showing a regular brain with no evidence of pathology.

**Figure 2:**
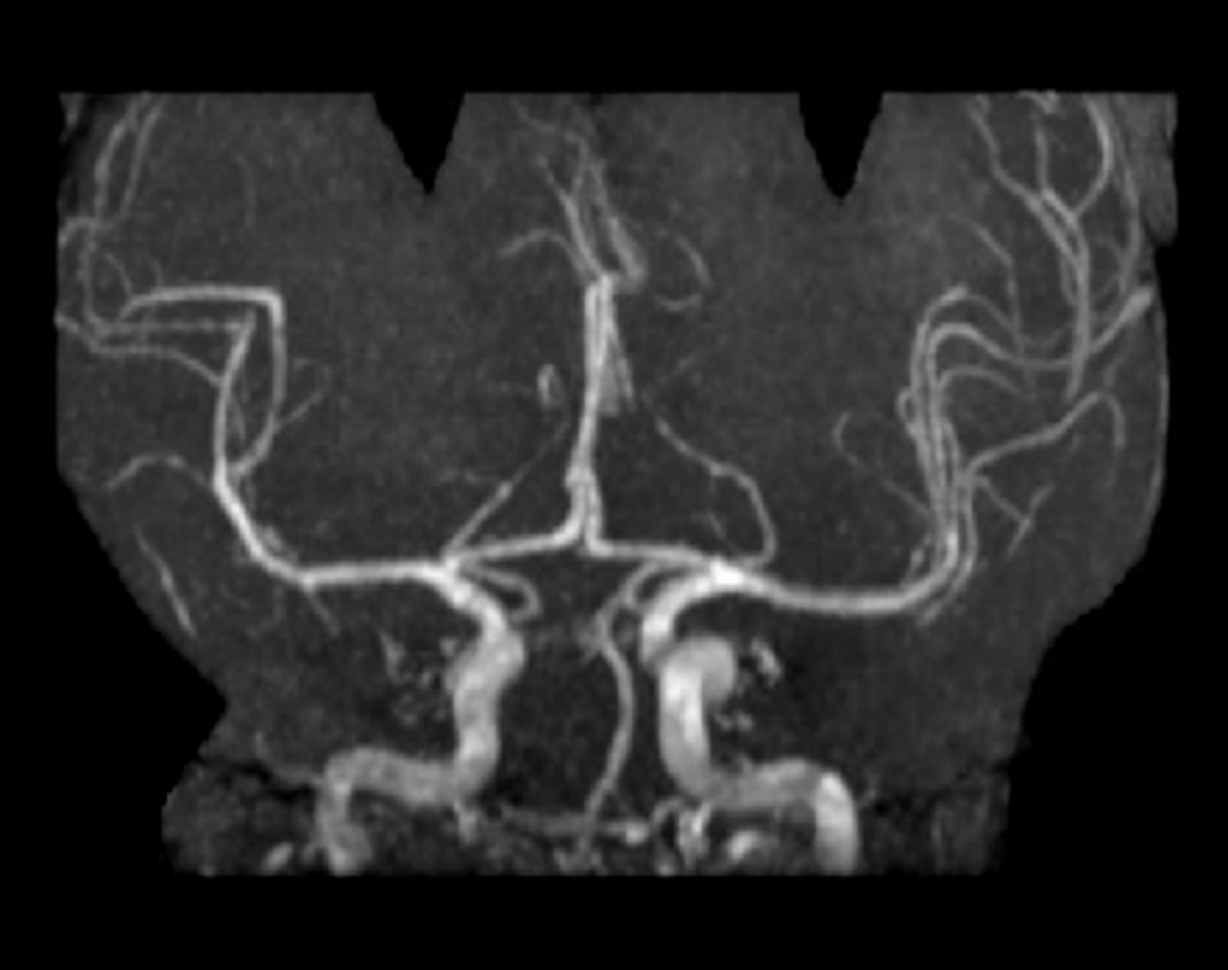
Magnetic Resonance Angiography Magnetic resonance angiography showing no evidence of pathology of the Circle of Willis.

Approximately one and a half months later, he was found unresponsive and catatonic in his car and readmitted to the hospital. His vital signs were temperature: 99 °F; pulse: 119/min; respiratory rate: 16/min; blood pressure: 112/85 mm Hg; and pulse oximetry on room air: 97%. His labs were WBC: 14.8 THOU/uL; hemoglobin: 17.2 g/dl; hematocrit: 52.3%; platelet count: 276 THOU/uL; sodium: 143 mmol/L; potassium: 3.9 mmol/L; creatinine: 0.7 mg/dL; glucose: 164 mg/dL; calcium: 9.6 mg/dL; AST: 22 U/L; and ALT: 14 U/L. During this stay, the patient appeared to be having latency in speech before responding to inquiries, but some days were significantly worse than others. He would be aware of his location but unable to explain why or for how long. He had a difficult time remembering names. He was still exhibiting abnormal involuntary movements around the mouth and extremities. Magnetic resonance imaging (MRI) was ordered to rule out stroke and vascular dementia; however, there was no acute intracranial pathology, as seen in Figure 1, and an unremarkable magnetic resonance angiogram (MRA) of the Circle of Willis, as seen in Figure 2.

**Figure 3:**
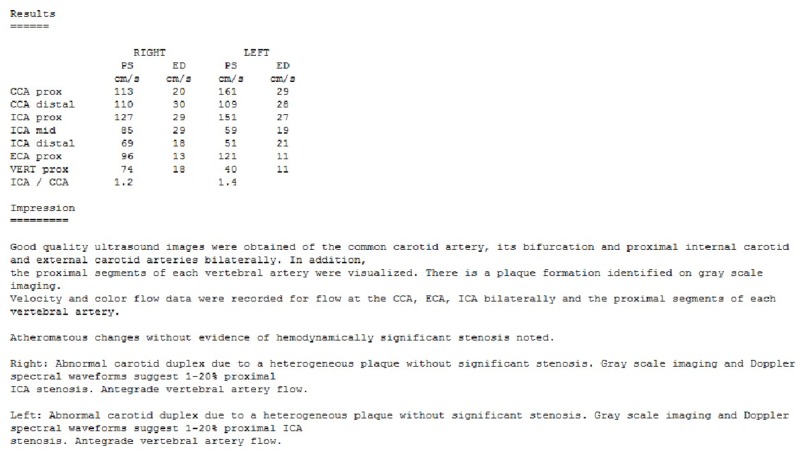
Carotid Duplex CCA, Common Carotid Artery; ICA, Internal Carotid Artery; ECA, External Carotid Artery; VERT, Vertebral Artery; PS, Peak Systolic Velocity; ED, End Diastolic Velocity; prox, proximal; mid, middle

His carotid ultrasound was unremarkable as well (Figure 3).

His history of late-onset auditory and visual hallucinations, sensitivity to neuroleptics, extrapyramidal symptoms, limited judgment, fluctuating cognition, and frequent emergency room (ER) visits due to altered mental status and deficiencies in the visual-spatial cognitive function may suggest Lewy body dementia. The patient was observed for a few days and was determined to have capacity before being discharged on zyprexa, ativan, and lithium.

## Discussion

Lewy body dementia is a very unique type of dementia that combines many psychiatric and neurologic components. Lewy body dementia derives its name from aggregates of alpha-synuclein in the brain, which became known as Lewy bodies [2]. These aggregates of alpha-synuclein are also seen in Parkinson’s disease. Lewy body dementia tends to follow a clinical pattern where visual hallucinations present before the onset of dementia and, thus, many cases of Lewy body dementia are underdiagnosed due to the lack of dementia symptoms during the first presentation of the disease.

The Diagnostic and Statistical Manual of Mental Disorders (DSM-V) has specific criteria for the diagnosis of Lewy body dementia. Since Lewy body dementia is only definitively diagnosed by a brain/autopsy biopsy, the DSM-V has two classifications of probable Lewy body dementia and possible Lewy body dementia. Probable Lewy body dementia is defined as having two or more core clinical features present or one core clinical feature present with one or more suggestive features also present [3]. Possible Lewy body dementia is defined as having only one core clinical feature present without suggestive features or one or more suggestive features present without any core clinical features present. The core clinical features of Lewy body dementia are waxing and waning cognition, visual hallucinations, and the development of extrapyramidal signs at least one year after cognitive decline becomes evident [4]. Suggestive features include rapid eye movement (REM) sleep behavior disorder and pronounced antipsychotic sensitivity. Other clinical signs with associations to Lewy body dementia are severe sensitivity to antipsychotic agents, hallucinations in other modalities, and syncope or other transient episodes of unresponsiveness [3].

Lewy body dementia is characterized by severe neuroleptic sensitivity. Unfortunately, Lewy body dementia may present with only visual hallucinations without clinical signs of dementia or extrapyramidal symptoms. In these scenarios, schizophrenia is often mistakenly diagnosed and treated with neuroleptic agents when there is actually an underlying Lewy body dementia. Caution must be advised when administering antipsychotics to patients with Lewy body dementia. If neuroleptic agents are used in cases of Lewy body dementia, current recommendations suggest the use of neuroleptic agents with a lower risk of developing extrapyramidal symptoms such as Zyprexa [5]. There are instances, as seen in this case report, where antipsychotics significantly decreased the patient's psychosis without significantly increasing his extrapyramidal symptoms. Zyprexa is an attractive candidate because it has reduced affinity for dopamine and acetylcholine receptors [5]. There is also supportive evidence in the current literature on Lewy body dementia that acetylcholinesterase inhibitors are an effective treatment option for neuropsychiatric and cognitive symptoms [1].

## Conclusions

This case report highlights the clinical progression of a case of probable Lewy body dementia with the documentation of multiple core clinical features, such as waxing and waning cognition, visual hallucinations, and extrapyramidal signs that the patient developed more than 12 months after his initial onset of hallucinations. The patient’s catatonia also supports the diagnosis of probable Lewy body dementia since this is a state of transient unresponsiveness. This presentation is unique because of the patient's predilection for auditory hallucinations and the success we had controlling his symptoms with zyprexa.
